# Effects of Weight-Bearing on Tibiofemoral, Patellofemoral, and Patellar Tendon Kinematics in Older Adults

**DOI:** 10.3389/fbioe.2022.820196

**Published:** 2022-04-14

**Authors:** Vasiliki Kefala, Azhar A. Ali, Landon D. Hamilton, Erin M. Mannen, Kevin B. Shelburne

**Affiliations:** ^1^ Department of Mechanical and Materials Engineering, University of Denver, Denver, CO, United States; ^2^ Stryker Orthopedics, Kalamazoo, MI, United States; ^3^ Department of Mechanical and Biomedical Engineering, Boise State University, Boise, ID, United States

**Keywords:** femur, tibia, patella, fluoroscopy, ligament, arthroplasty

## Abstract

Quantification of natural knee kinematics is essential for the assessment of joint function in the diagnosis of pathologies. Combined measurements of tibiofemoral and patellofemoral joint kinematics are necessary because knee pathologies, such as progression of osteoarthritis and patellar instability, are a frequent concern in both articulations. Combined measurement of tibiofemoral and patellofemoral kinematics also enables calculation of important quantities, specifically patellar tendon angle, which partly determines the loading vector at the tibiofemoral joint and patellar tendon moment arm. The goals of this research were to measure the differences in tibiofemoral and patellofemoral kinematics, patellar tendon angle (PTA), and patellar tendon moment arm (PTMA) that occur during non-weight-bearing and weight-bearing activities in older adults. Methods: High-speed stereo radiography was used to measure the kinematics of the tibiofemoral and patellofemoral joints in subjects as they performed seated, non-weight-bearing knee extension and two weight-bearing activities: lunge and chair rise. PTA and PTMA were extracted from the subject’s patellofemoral and tibiofemoral kinematics. Kinematics and the root mean square difference (RMSD) between non-weight-bearing and weight-bearing activities were compared across subjects and activities. Results: Internal rotation increased with weight-bearing (mean RMSD from knee extension was 4.2 ± 2.4° for lunge and 3.6 ± 1.8° for chair rise), and anterior translation was also greater (mean RMSD from knee extension was 2.2 ± 1.2 mm for lunge and 2.3 ± 1.4 mm for chair rise). Patellar tilt and medial–lateral translation changed from non-weight-bearing to weight-bearing. Changes of the patellar tendon from non-weight-bearing to weight-bearing were significant only for PTMA. Conclusions: While weight-bearing elicited changes in knee kinematics, in most degrees of freedoms, these differences were exceeded by intersubject differences. These results provide comparative kinematics for the evaluation of knee pathology and treatment in older adults.

## 1 Introduction

Quantification of natural knee kinematics is essential for the assessment of joint function in the diagnosis of pathologies. Combined measurements of tibiofemoral and patellofemoral joint kinematics are necessary because knee pathologies, such as progression of osteoarthritis and patellar instability, are a frequent concern in both articulations. *In vitro* studies have documented how alteration in tibiofemoral joint translation and rotation can change patellofemoral joint function (Van Kampen and Huiskes 1990; Hefzy et al., 1992; Li et al., 2004). Likewise, patellofemoral dysfunctions, such as patellar maltracking, have been linked with a combined abnormal motion of the tibia and patella relative to the femur (Fulkerson et al., 1990; Bull et al., 2002). Furthermore, complications following treatments at the tibiofemoral joint, namely, anterior cruciate ligament repair, may cause scarring that produces a relative shortening of the patellar tendon (Ahmad et al., 1998), and procedures including high tibial osteotomy and total knee arthroplasty may alter the joint line and create patella baja or pseudo-patella baja (Closkey and Windsor 2001; Grelsamer 2002). Combined measurement of tibiofemoral and patellofemoral kinematics also enables calculation of important quantities, specifically the patellar tendon angle (PTA), which partly determines the loading vector at the tibiofemoral joint (Gill and O’connor 1996; Miller et al., 1998; Rees et al., 2005; Stagni et al., 2010), and patellar tendon moment arm (PTMA), which has been used as a surrogate measurement for the efficiency of the quadriceps (Maganaris et al., 2001). Yet, reported measurements of both tibiofemoral and patellofemoral motion of the healthy living knee during activities of daily living are very rare. Studies have reported precision kinematics for the tibiofemoral joint (Lafortune et al., 1992; Andriacchi and Dyrby 2005; Li et al., 2005; Kefala et al., 2017) and the patellofemoral joint (Schutzer et al., 1986; Koh et al., 1992; Kefala et al., 2021a), yet only a few have investigated the combined motion of tibiofemoral and patellofemoral joints in healthy living subjects (Li et al., 2007; Seisler and Sheehan 2007; Thomeer et al., 2021).

Motion of the tibiofemoral and patellofemoral joints likely change as subjects move from non-weight-bearing to weight-bearing having implications for surgical repairs to the unloaded knee. Knowledge of how knee kinematics changes with demand can provide kinematic targets for treatments that seek to restore normal function. Myers et al. (2012) measured an increase in anterior displacement of the tibia relative to the femur as demand (defined as increasing knee extensor moment) on the knee increased. Furthermore, weight-bearing induces strain in repaired ligaments (Fleming et al., 2001; Kernkamp et al., 2018) and tracking of the patella in the femoral groove (Draper et al., 2011). However, treatments that seek to restore normal weight-bearing motion to the knee might be confounded by patient-specific knee motion that may routinely fall outside population norms (Hume et al., 2018). Hume et al. (2018) demonstrated that spline representations of knee kinematics may adequately predict knee motion of a group but poorly predict individual kinematics.

Documenting knee kinematics in older adults is necessary for understanding the changes that occur with pathology. Osteoarthritis of the patellofemoral and tibiofemoral joints and surgical repairs including knee arthroplasty are common in this age group. Several prior studies have evaluated young healthy, osteoarthritis, and total knee arthroplasty subjects and shown important differences between knee kinematics across these cohorts. With a few notable exceptions (Fukagawa et al., 2012; Kefala et al., 2021b), these studies routinely compare older adults with osteoarthritis or knee arthroplasty to young healthy controls. The motion of younger subjects may not be representative of the age range associated with knee pain, osteoarthritis, and arthroplasty. Older adults tend to use unique movement strategies while stepping down or turning and pivoting (Fuller et al., 2007; Samuel et al., 2011; Chen et al., 2013). Although these studies and others (Pfeiffer et al., 2014) have noted that aging has an impact on knee kinematics measured using marker-based motion capture, few studies have examined the small translations and rotations of the knee in older adults with no history of knee pathology, and none have reported kinematics for both the tibiofemoral and patellofemoral articulations.

The goals of this research were to 1) quantify differences in tibiofemoral and patellofemoral kinematics, and patellar tendon angle and moment arm that occur from non-weight-bearing to weight-bearing in older adults; 2) determine if changes in tibiofemoral kinematics correspond with changes in patellofemoral kinematics; and 3) examine whether these differences in kinematics were variable between individuals.

## 2 Methods

### 2.1 Participants

Seventeen healthy individuals with no history of injuries or surgeries to the lower limbs (8F/9M, age = 66.0 ± 7.9 years old, body mass = 75.7 ± 20.5 kg, body mass index = 26.4 ± 5.1 kg/m^2^, height = 166.8 ± 10.9 cm) provided informed consent and participated in the University of Denver Institutional Review Board-approved study.

### 2.2 Protocol

High-speed stereo radiography (HSSR) was used to measure the kinematics of the tibiofemoral and patellofemoral joints in each subject’s dominant knee (Nha et al., 2008; Ivester et al., 2015). HSSR captures two radiographic views to enable three-dimensional tracking of the bones in the knee (Ivester et al., 2015). The HSSR system is composed of two matching custom radiography systems with 40-cm (16-in)-diameter image intensifiers integrated with high-speed, high-definition (1080 × 1080) digital cameras (Ivester et al., 2015). The accuracy of the HSSR system for tracking the tibia, femur, and patella was confirmed by comparison to measurements made in bones with implanted tantalum beads (Ivester et al., 2015). The average translational tracking error of the patella was 0.1 ± 0.05 mm, and the average rotational tracking error was 0.4 ± 0.2°. Similarly, for the femur, the average translational tracking error was 0.1 ± 0.12 mm and the average rotational tracking error was 0.1 ± 0.13°. Thus, the expected tracking error was much less than the observed motion of the tibia and patella relative to the femur in the current study. Studies using similar dual-plane radiography equipment and methods recorded patella tracking accuracy of 0.46–0.60 mm in translation and 0.99°–1.14° in rotation and femur tracking accuracy of 0.40–0.70 mm in translation and 0.50°–0.80° in rotation (Bey et al., 2008; Ohnishi et al., 2010). All activities were captured at 50 frames/second and obtained with pulsed radiography (pulse width 750 μs, 60 kV, and 63 mA). The effective dose of this experiment as a whole was 0.28 mSv, which was the combined effective dose from computed tomography (CT) (0.16 mSv (Biswas et al., 2009)) and from radiography (0.12 mSv, PCXMC, STUK, Helsinki, Finland).

### 2.3 Data Collection

**FIGURE 1:**
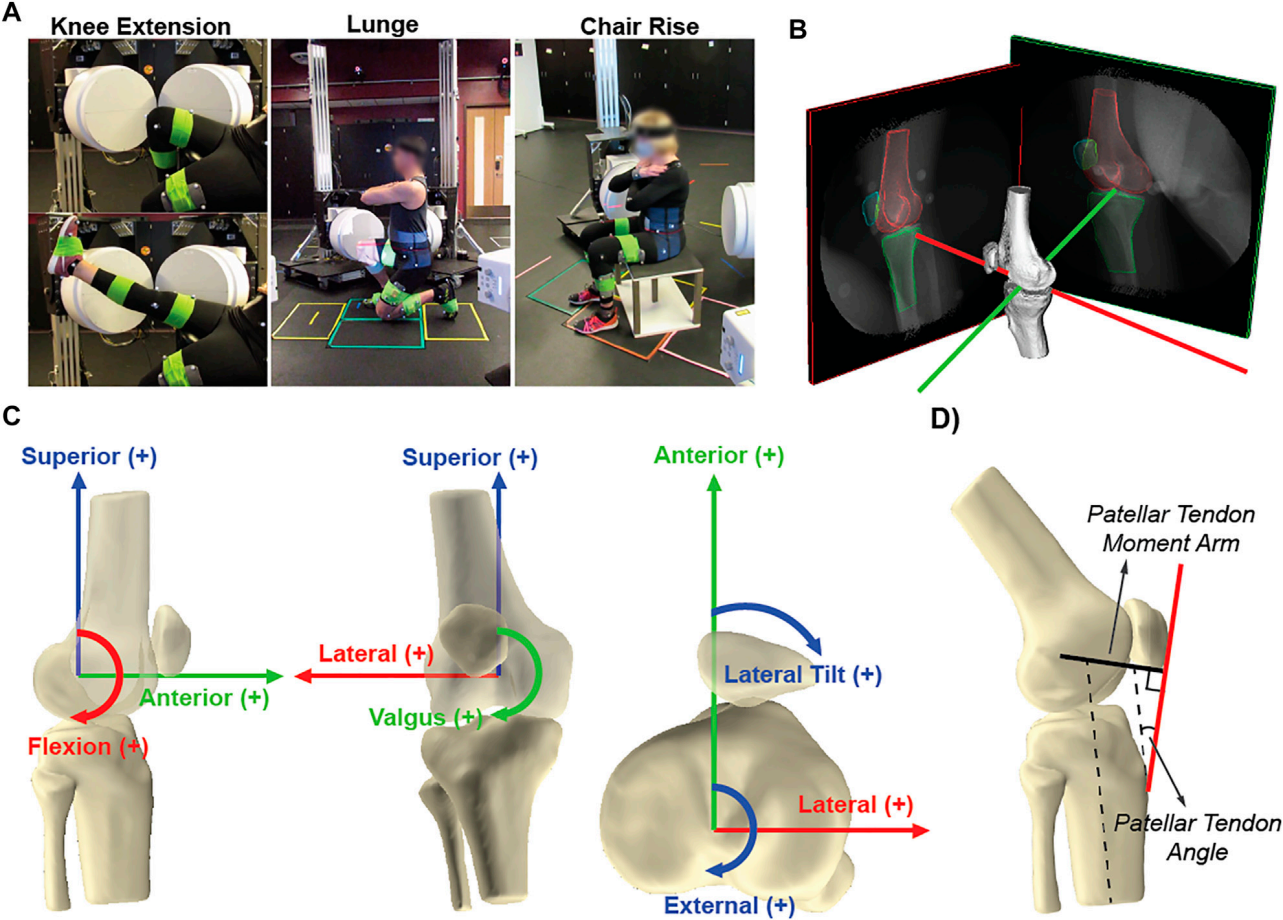
**(A)** Participants performed seated knee extension, rising from a chair, and lunge with their dominant knee in view of the radiography system, and **(B)** Representative bone tracking of the femur, patella, and tibia in high-speed stereo radiography (HSSR). **(C)** origin of the femoral coordinate system for each subject was defined by fitting a cylinder to the medial and lateral posterior condyles, with the center placed at the trochlea. The coordinate system of the tibia and patella was assigned coincident with the femoral coordinate system at full extension; **(D)** patellar tendon angle (PTA) was taken as the line between the inferior pole of the patella, the tibial tuberosity, and the long axis of the tibia, and moment arm (PTMA) was calculated as the perpendicular distance from the patellar tendon to the instantaneous axis of rotation of the tibiofemoral joint.

Participants performed three activities: 1) knee extension in which the individuals were seated and slowly extended their knee from high flexion to full extension (seated knee extension); 2) weight-bearing deep knee bend (lunge); 3) and standing up from a chair without arm support (chair rise) (Figure 1). One subject did not complete the lunge; therefore, sixteen subjects were reported for that activity. Following the laboratory data collection, a static bone CT scan with a slice thickness of 1.0 mm was obtained for each subject’s dominant knee.

### 2.4 Data Processing

Three-dimensional models of the distal femur, proximal tibia, and patella bones were reconstructed from the CT data using ScanIP (Simpleware Inc.). Positions of the three-dimensional bone models were matched to the two-dimensional stereo radiography images to quantify the translational and rotational pose of the tibia and patella relative to the femur (Autoscoper, Brown University (Brainerd et al., 2010)).

The femoral coordinate system was defined by first fitting a cylinder to the sagittal projection of the articular surface of the medial and lateral posterior femoral condyles (Figure 1) (Torry et al., 2011; Hancock et al., 2013; Kefala et al., 2017). The coordinate system was located on the medial–lateral axis of the cylinder and centered at the trochlea. The superior–inferior axis was placed normal to the medial–lateral axis and oriented superiorly and parallel to the anatomical axis of the femur, using the centroids of the distal and proximal shafts. The anterior–posterior axis was oriented anteriorly and defined as the cross-product of the medial–lateral and superior–inferior axes. The coordinate system of the tibia and patella was assigned coincident with the femoral coordinate system during non-weight-bearing full extension (Kefala et al., 2021a) to reveal changes from each participant’s neutral pose (Woo et al., 1999). The motion of the tibia and patella was described relative to the femur (Grood and Suntay 1983) and filtered using a 4^th^-order low-pass Butterworth filter with a cutoff frequency of 2 Hz for all three activities. The contact points of the femur on the tibial plateau were estimated with tibiofemoral low-point kinematics (Asano et al., 2001; Komistek et al., 2003; Li et al., 2005), calculated by finding the most distal points of the femoral condyles relative to the tibia as a function of flexion. Low-point kinematics of the femur relative to the medial/lateral tibial plateau was used to illustrate notable individual differences.

### 2.5 Data Analyses

Six kinematic quantities were reported with respect to tibial flexion angle: tibial external rotation (TIE), valgus rotation (TVV), anterior translation (TAP), patellar flexion (PFE), tilt (PTilt), and medial translation (PML). In addition, the patellar tendon angle (PTA) was taken as the line between the inferior pole of the patella, the tibial tuberosity, and the long axis of the tibia. Patellar tendon moment arm (PTMA) was calculated as the perpendicular distance from the patellar tendon to the instantaneous axis of rotation of the tibiofemoral joint. The patella tendon was defined as a straight line from the patella apex to the tibial tuberosity, and the instantaneous axis of rotation was calculated using the equations described by Spoor and Veldpaus (1980). Root-mean-square difference (RMSD) was used to quantify the differences between the non-weight-bearing seated knee extension and weight-bearing, lunge and chair rise, for each individual and averaged across subjects (average RMSD). A cubic spline was used to interpolate data in 1° increments from minimum to maximum knee angles for each degree of freedom (DOF) (lambda = 0.01) to provide a direct comparison between activities. The RMSD values between knee extension and the weight-bearing trials were calculated across an angle-matched flexion range (differed for each participant) for TIE, TVV, TAP, PFE, PTilt, PML, PTA, and PTMA. In addition, RMSD values were scaled by the total excursion of each DOF during the task. Scaled RMSD values provided context by revealing differences that exceed the range of motion of that DOF (Akbarshahi et al., 2010; Stagni et al., 2010; Hume et al., 2018). Accordingly, a scaled value of a RMSD greater than 1.0 indicated a substantial change in RMSD that exceeded the range of motion during the activity. Visual inspection of the distribution for RMSD values and quantile–quantile plots was performed to ensure a normal distribution exists for each activity and DOF. Additionally, 95% confidence intervals of the average RMSD point estimates were calculated between weight-bearing and non-weight-bearing activities for each activity.

## 3 Results

### 3.1 Tibiofemoral Kinematics

**FIGURE 2:**
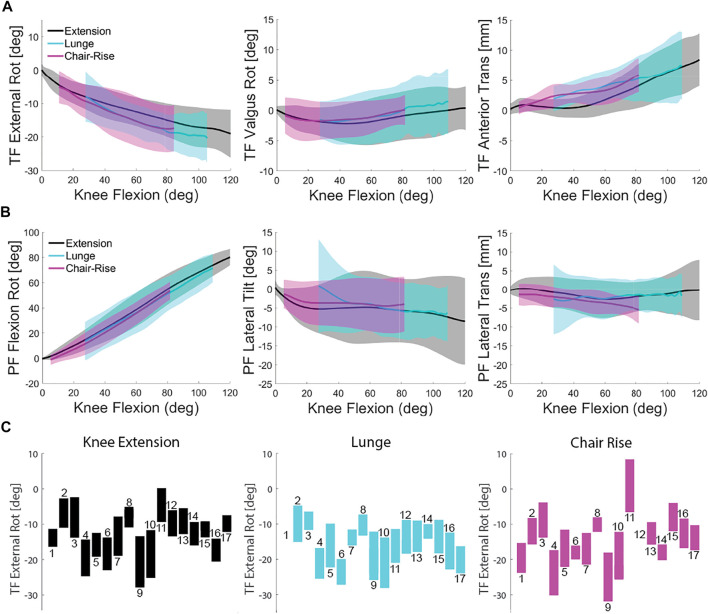
**(A)** Comparison of tibia kinematics relative to the femur showing average and one standard deviation of the three activities. **(B)** Comparison of patella kinematics relative to the femur showing average and one standard deviation of the three activities. **(C)** TIE range of motion for each subject for all three activities (lunge was not performed by Subject 1).

On average, tibial rotation (TIE) was more internal during weight-bearing (Figure 2A). Average TIE was −11.0 ± 3.6° for knee extension compared with lunge (-17.1 ± 5.4°), and chair rise (−11.9 ± 5.2°). Table 1 summarizes the results for tibiofemoral kinematics for the entire range of motion for each activity and for the angle-matched flexion range (Seated knee extension and Lunge: 40° to 90°; seated knee extension and chair rise: 10° to 70°). Mean RMSD value for non-weight-bearing (seated knee extension) to weight-bearing was 4.2 ± 2.4 mm [3.0, 5.5] for lunge and 3.6 ± 1.8 mm [2.7, 4.6] for chair rise (Table 2). With 95% confidence, the true mean RMSD did not include 0 in lunge or chair rise, indicating a significant difference in RMSD between weight-bearing and non-weight-bearing (mean scaled RMSD- 0.5 and 0.3, for lunge and chair rise, Table 2). Substantial variation in TIE was observed between subjects that remained consistent across activities (Figure 2C).

**TABLE 1:** Minimum, maximum, average, and range of motion kinematics for the tibia relative to the femur during the knee extension, lunge, and chair rise. Kinematics is reported for the full range of knee flexion, angle-matched flexion range from 10° to 70° for comparison of knee extension to chair rise, and angle-matched flexion range from 40° to 90° for comparison of knee extension to lunge.

	TF external rotation [°]			TF valgus rotation [°]			TF anterior translation [mm]		
	Full range	10°–70°	40°–90°	Full range	10°–70°	40°–90°	Full range	10°–70°	40°–90°
Knee extension									
Minimum	−19.9 ± 6.7	−14.1 ± 5.0	−16.7 ± 6.1	−2.9 ± 2.7	−2.3 ± 3.5	−1.9 ± 3.9	−0.8 ± 1.0	−0.4 ± 1.2	0.3 ± 1.8
Maximum	0.4 ± 2.0	−5.0 ± 2.5	−10.0 ± 4.5	2.7 ± 3.3	0.4 ± 3.1	0.4 ± 4.1	9.0 ± 4.4	3.0 ± 2.2	5.1 ± 3.4
Average	−11.0 ± 3.6	−9.6 ± 3.6	−13.4 ± 5.0	−0.4 ± 3.0	−1.1 ± 3.4	−0.9 ± 4.0	3.0 ± 2.2	0.9 ± 1.5	2.4 ± 2.6
ROM	20.2 ± 6.8	9.0 ± 4.5	6.5 ± 3.5	5.6 ± 2.5	2.7 ± 1.7	2.3 ± 1.3	9.7 ± 3.6	3.5 ± 1.4	4.8 ± 1.9
Lunge									
Minimum	−22.3 ± 6.6		−19.8 ± 5.4	−2.3 ± 3.8		−2.1 ± 4.0	1.3 ± 2.1		1.9 ± 2.0
Maximum	−7.5 ± 7.3		−12.6 ± 4.1	2.9 ± 5.0		1.6 ± 4.9	7.8 ± 4.6		5.6 ± 2.5
Average	−17.1 ± 5.4		−16.5 ± 4.5	0.4 ± 4.8		−0.3 ± 4.5	5.1 ± 3.3		3.8 ± 2.1
ROM	14.8 ± 8.5		7.2 ± 3.5	5.2 ± 2.2		3.7 ± 1.6	6.5 ± 3.4		3.7 ± 1.6
Chair rise									
Minimum	−18.6 ± 7.2	−16.5 ± 6.5		−2.4 ± 3.3	−2.4 ± 3.3		0.6 ± 1.2	1.0 ± 0.9	
Maximum	−4.6 ± 4.6	−5.8 ± 4.5		1.0 ± 3.1	−0.2 ± 3.2		6.4 ± 2.7	4.9 ± 2.0	
Average	−11.9 ± 5.2	−10.8 ± 5.5		−1.0 ± 3.2	−1.4 ± 3.2		3.2 ± 1.5	2.9 ± 1.4	
ROM	14.1 ± 7.5	10.7 ± 4.7		3.4 ± 1.4	2.2 ± 1.3		5.8 ± 2.9	3.9 ± 1.9	

**TABLE 2:** Root-mean-square difference (RMSD) between the non-weight-bearing and weight-bearing activities averaged across all subjects. Scaled RMSDs were calculated as the RMSD divided by the ROM in each degree of freedom. Green highlights scaled RMSD values of 0.5 or greater.

RMSD	TF VV degrees scaled	TF IE degrees scaled	TF AP mm scaled	PF FE degrees scaled	PF Tilt degrees scaled	PF ML mm scaled	PFMA mm scaled	PTA degrees scaled
Lunge	1.8	4.2	2.2	4.9	5.0	4.2	4.0	2.6
	0.4	0.5	0.5	0.1	0.7	0.7	0.3	0.2
Chair rise	2.0	3.6	2.3	4.3	5.3	4.6	4.8	2.7
	0.7	0.3	0.5	0.1	0.7	0.9	0.3	0.2

Most of the subjects remained near neutral alignment or varus (TVV, Figure 2A) throughout the seated knee extension, lunge, and chair rise (average −0.4 ± 3.0°, 0.4 ± 4.8°, and −1.0 ± 3.2°, respectively, Table 1). The change in TVV from non-weight-bearing to weight-bearing was small but significantly different (mean RMSD 1.8 ± 0.9° [1.3, 2.2] and 2.0 ± 0.9° [1.5, 2.4] in lunge and chair rise, respectively). Scaled RMSD values were less than 0.5 for ten subjects due to the overall small excursion of TVV (mean scaled RMSD 0.4 and 0.7, for lunge and chair rise, Table 2), but these differences likely are not clinically meaningful.

Tibial anterior translation (TAP, Figure 2A) during the lunge and chair rise was greater than during knee extension (3.0 ± 2.2 mm, 5.1 ± 3.3 mm, and 3.2 ± 1.5 mm for knee extension, lunge, and chair rise, respectively, Table 1). Mean RMSD values were 2.2 ± 1.2 mm [1.6, 2.8] for lunge and 2.3 ± 1.4 mm [1.6, 3.0] for chair rise with scaled RMSD of 0.5 and 0.5, respectively (Table 2). With 95% confidence, the true mean RMSD did not include 0 in lunge or chair rise, indicating significant difference in RMSD between weight-bearing and non-weight-bearing.

**FIGURE 3:**
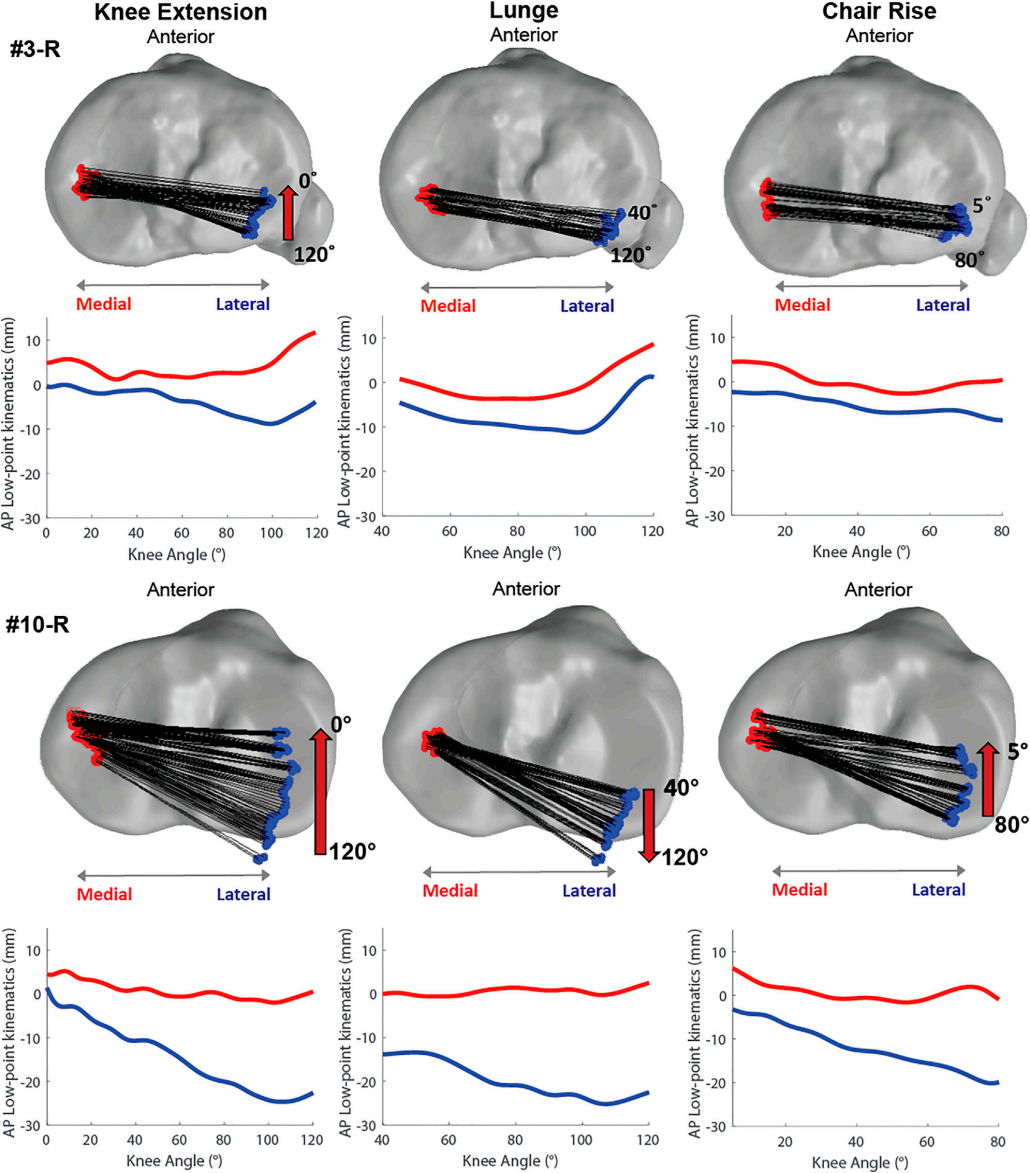
Illustrative low-point kinematics for two subjects during the three activities showing consistent tibial rotation across activities with distinct subject differences.

Low-point kinematics indicated a more medial pivot (greater overall TAP translation than medial TAP translation) for ten of the subjects for all three activities (e.g., Subject 10, Figure 3). However, four subjects displayed a medial pivot during seated knee extension that was not observed during the two weight-bearing activities (e.g., Subject 3, Figure 3), and the remaining three subjects displayed no apparent pivot during activities.

### 3.2 Patellofemoral Kinematics

All three activities demonstrated patellofemoral flexion closely tied to the knee flexion angle (PFF, Figure 2B). Average PFF was 36.9 ± 7.5° for knee extension compared with lunge (50.0 ± 12.9°) and chair rise (25.4 ± 5.9°). Table 3 summarizes the results for patellofemoral kinematics for the entire range of motion for each activity. The mean RMSD values from weight-bearing to non-weight-bearing were 4.9 ± 4.1° [2.7, 7.1] for lunge and 4.3 ± 1.8° [3.4, 5.3] for chair rise (scaled RMSD 0.1 and 0.1, respectively, Table 2). With 95% confidence, the true mean RMSD did not include 0 in lunge or chair rise, indicating a significant difference in RMSD between weight-bearing and non-weight-bearing.

**TABLE 3:** Minimum, maximum, average, and range of motion kinematics for the patella relative to the femur during knee extension, lunge, and chair rise. Kinematics is reported for the full range of knee flexion, angle-matched flexion range from 10° to 70° for comparison of knee extension to chair rise, and angle-matched flexion range from 40° to 90° for comparison of knee extension to lunge.

	PF flexion [°]			PF lateral tilt [°]			TF anterior translation [mm]		
	Full range	10°–70°	40°–90°	Full range	10°–70°	40°–90°	Full range	10°–70°	40°–90°
Knee extension									
Minimum	−0.6 ± 1.1	4.3 ± 2.2	24.0 ± 4.6	−10.7 ± 7.4	−7.6 ± 6.4	−7.2 ± 7.6	−4.6 ± 4.6	−3.3 ± 3.9	−3.1 ± 4.5
Maximum	82.3 ± 6.8	46.7 ± 6.3	61.8 ± 5.8	2.4 ± 4.8	−1.5 ± 6.2	−3.3 ± 8.2	3.5 ± 3.1	0.8 ± 2.9	−0.6 ± 3.8
Average	36.9 ± 7.5	22.0 ± 4.1	42.9 ± 5.6	−5.0 ± 6.2	−4.8 ± 6.1	−5.2 ± 8.0	−0.8 ± 3.1	−1.3 ± 3.4	−1.9 ± 4.1
ROM	82.9 ± 6.5	42.4 ± 5.8	37.8 ± 3.3	13.1 ± 6.3	6.1 ± 3.2	3.9 ± 2.5	8.0 ± 4.2	4.1 ± 2.7	2.5 ± 1.6
Lunge									
Minimum	−9.7 ± 8.1		23.4 ± 4.6	−7.2 ± 7.3		−2.5 ± 7.4	−6.7 ± 6.5		−4.6 ± 4.3
Maximum	70.0 ± 12.6		60.0 ± 7.0	1.9 ± 10.2		−8.5 ± 8.3	1.9 ± 5.1		0.6 ± 4.9
Average	50.0 ± 12.9		42.8 ± 7.0	−4.7 ± 7.4		−5.3 ± 8.0	−2.3 ± 4.2		−2.1 ± 4.2
ROM	56.3 ± 13.5		34.5 ± 4.6	11.6 ± 8.3		6.0 ± 3.0	8.6 ± 5.7		5.1 ± 3.1
Chair rise									
Minimum	−6.2 ± 6.2	4.1 ± 5.5		−5.9 ± 6.7	−5.1 ± 6.4		−7.0 ± 4.1	−6.3 ± 4.0	
Maximum	55.8 ± 8.1	43.3 ± 5.1		2.5 ± 5.6	1.6 ± 6.5		0.1 ± 3.9	−1.0 ± 4.6	
Average	25.4 ± 5.9	19.4 ± 5.7		−2.2 ± 6.2	−1.7 ± 6.6		−3.3 ± 3.9	−3.7 ± 4.4	
ROM	54.4 ± 9.8	39.1 ± 6.9		8.7 ± 3.5	6.6 ± 2.9		7.1 ± 3.9	5.3 ± 2.9	

Patellar medial-lateral tilt (PTilt, Figure 2B) was medial (average: −5.0 ± 6.2°, knee extension: −4.7 ± 7.4°, lunge: and −2.2 ± 6.2° for chair rise, Table 3). Individually, most subjects consistently maintained either a medial or lateral tilt throughout the activities. The mean RMSD values were 5.0 ± 2.9° [3.5, 6.6] for lunge and 5.3 ± 4.4° [3.0, 7.6] for chair rise, which was similar to the ROM (average scaled RMSD was 0.7 in lunge and 0.7 in chair rise). With 95% confidence, the true mean RMSD did not include 0 in lunge or chair rise, indicating significant difference in RMSD between weight-bearing and non-weight-bearing.

Patellar medial–lateral translation (PML, Figure 2B) was medial in most subjects for knee extension, lunge, and chair rise (average: −0.8 ± 3.1 mm, −2.3 ± 4.2 mm, and −3.3 ± 3.9 mm, respectively, Table 3). Individually, some of the subjects’ patellae remained in a more lateral position. The mean RMSD was 4.2 ± 2.4 mm [2.9, 5.4] for lunge and 4.6 ± 3.5 mm [2.7, 6.4] for chair rise, which nearly matched the ROM (average scaled RMSD was 0.7 in lunge and 0.9 in chair rise, Table 2). With 95% confidence, the true mean RMSD did not include 0, indicating a significant difference in RMSD between weight-bearing and non-weight-bearing.

### 3.3 Patellar Tendon Angle and Moment Arm

**FIGURE 4:**
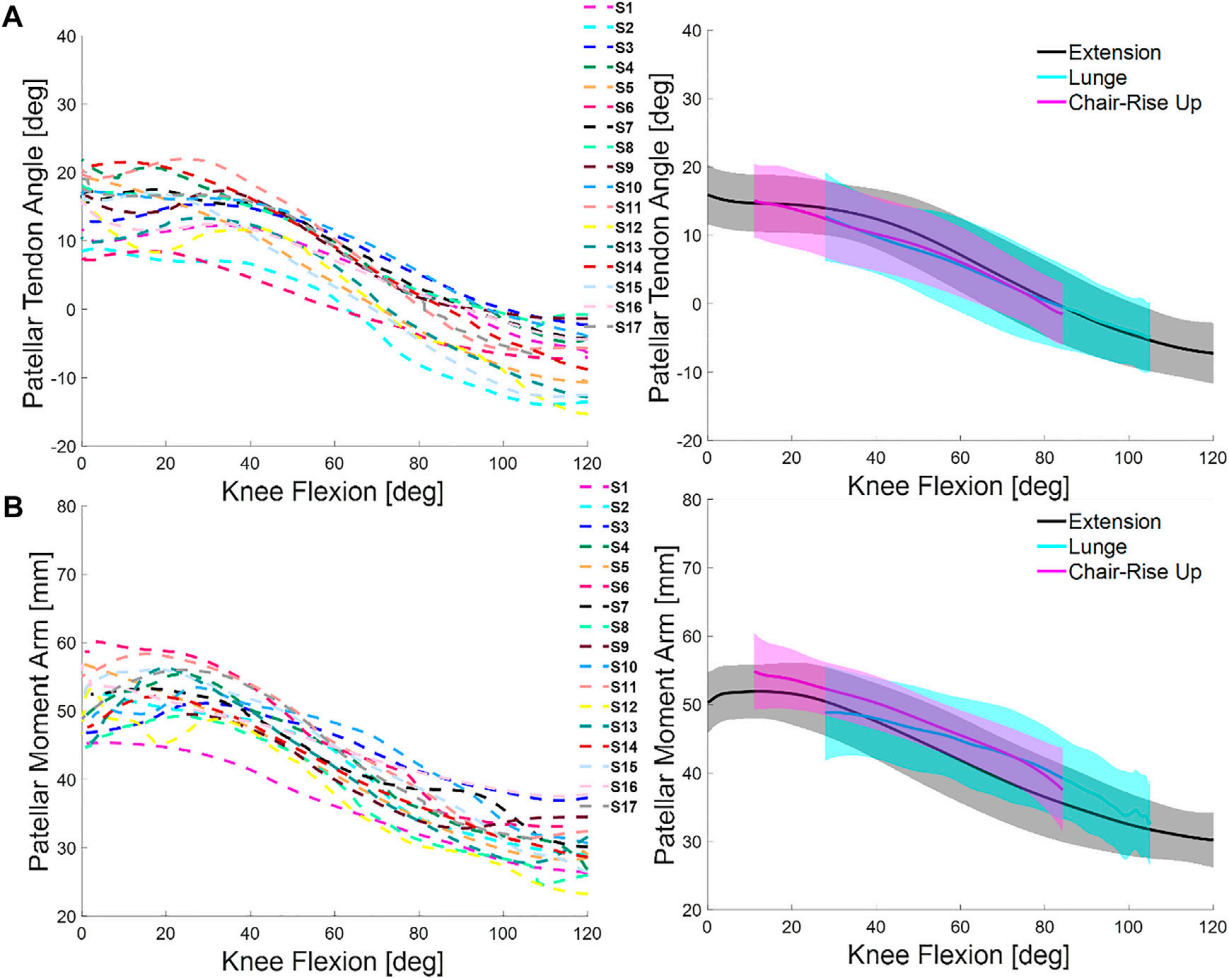
**(A)** Comparison of patellar tendon angle (PTA) across subjects during the seated knee extension (left) and comparison of average and one standard deviation across activities (right). **(B)** Comparison of patellar tendon moment arm (PTMA) across subjects during the seated knee extension (left) and comparison of average and one standard deviation across activities (right).

All subjects demonstrated consistent PTAs across the activities, with the highest PTAs at low knee flexion angles (e.g., average 11.7 ± 4.5° at full extension during seated knee extension) and the lowest PTAs at maximum knee flexion (e.g., average 0.1 ± 3.6° at 80° during seated knee extension, Figure 4A). Average PTA were 6.2 ± 3.5°, 0.9 ± 5.5°, and 8.4 ± 4.6° for seated knee extension, lunge, and chair rise, respectively. Patellar-tendon angle for each subject remained consistent during activities as demonstrated by low RMSD (2.6 ± 1.4° [1.9, 3.3] for lunge and 2.7 ± 1.1° [2.1, 3.3] for chair rise) and scaled RMSD (0.2 for lunge and 0.2 for chair rise; Table 2). Across individuals, the differences in PTA were large compared with the differences between activities (Figure 4A).

Across activities, patellar tendon moment arm (PTMA) was highest at full extension (e.g., 51.4 ± 2.5 mm during seated knee extension) and lowest at high knee flexion angles (e.g., 38.7 ± 1.7 mm at 80° during seated knee extension, Figure 4B). Average PTMA was 42.4 ± 3.8 mm, 39.6 ± 5.5 mm, and 48.1 ± 3.7 mm, for knee extension, lunge, and chair rise, respectively (Figure 4B). Large differences were observed between individuals (Figure 4B). The mean RMSD values were 4.0 ± 2.2° [2.8, 5.1] for lunge and 4.8 ± 2.9° [3.2, 6.3] for chair rise, which was similar to the ROM (average scaled RMSD was 0.3 in lunge and 0.3 in chair rise; Table 2). With 95% confidence, the true mean RMSD did not include 0 in lunge or chair rise, indicating significant difference in RMSD between weight-bearing and non-weight-bearing.

## 4 Discussion

Quantification of natural knee kinematics is important for assessment of joint function in the diagnosis of pathologies, such as progression of osteoarthritis and patellar instability, and for evaluation of outcomes following conservative or surgical treatment. In this study, a high-speed stereo radiography system was used to investigate multiple DOFs of tibiofemoral and patellofemoral kinematics in healthy, older adults during a non-weight-bearing seated knee extension and two weight-bearing activities, lunge and chair rise. Tibia motion, relative to the femur, was consistent in trend during the seated knee extension, lunge, and chair rise with the exceptions of increased internal rotation and anterior translation during weight-bearing activities. The axis of tibial rotation was on the medial side of the knee (medial pivot) for all three activities, with notable intersubject differences in the amount of rotation (Figure 2C). Average patella ROM during the lunge and chair rise activities was within the ROM of the knee extension, demonstrating consistent tracking of the patella; however, moving from non-weight-bearing to weight-bearing created changes in patellar tilt and medial–lateral translation. Although changes in PTA and PTMA from non-weight-bearing to weight-bearing were small, substantial individual differences were measured.

Like prior studies, general trends in tibia motion relative to the femur were similar for all subjects across activities. Tibial kinematics during seated knee extension agreed with Myers et al. (2012) where peak internal rotation for unweighted knee extension was 14.5 ± 7.7° at 90° of knee flexion (cf. 16° in Figure 2A) and peak anterior translation was 2.6 ± 2.1 mm (cf. 4 mm in Figure 2A). In further agreement with their findings and those of Thomeer et al. (2021), weight-bearing caused changes in kinematics. In the current study, internal rotation and anterior translation increased during lunge and chair rise. However, the average RMSD values for TIE (4.2 and 3.6 for lunge and chair rise) were less than the TIE standard deviation of the subjects (Table 1 and Figure 2A), suggesting tibial rotation was more sensitive to subject differences than weight-bearing. Furthermore, the axis of tibial rotation was located on the medial side of the knee during all activities for the majority of the subjects lending support to the concept of medial pivot in total knee arthroplasty (Shimmin et al., 2015); however, the amount of internal rotation varied widely between subjects (Figure 2C), with three subjects showing no evidence of medial pivot (Figure 3). As expected from prior reports, TAP translation increased with weight-bearing for most subjects (Figure 2A) (Myers et al., 2012); however, the impact was moderate relative to the ROM of TAP (e.g., scaled RMSD values were 0.5 for lunge and chair rise). Conversely, TVV rotation was consistent for all three activities as demonstrated by the small average standard deviation (Table 1 and Figure 2A) and the low RMSD values compared to the other DOFs (Table 2). These results agreed with several studies that have measured small amounts of TVV during activities (Myers et al., 2012; Kefala et al., 2017; Thomeer et al., 2021). However, TVV was also affected by weight-bearing as demonstrated by the scaled RMSD values that showed these changes were on the order of the natural TVV range of motion (e.g., scaled RMSD of 0.7 for chair rise).

As shown in prior studies (Hefzy et al., 1992; Li et al., 2007; Thomeer et al., 2021), there was a strong correlation between patellar flexion and tibiofemoral flexion both on average and across individuals (e.g., average *R* ^2^ = 0.96 for knee extension). Moreover, weight-bearing had the smallest effect on patellar flexion with the lowest scaled RMSD of all the kinematics measured (Table 2). In contrast, weight-bearing changed both PTilt and PML. The change in PTilt with weight-bearing was medial in some subjects and lateral in others and as great as the unweighted ROM leading to scaled RMSD values of 0.7 for lunge and chair rise. Greater PML was observed for the chair rise compared to seated knee extension and lunge. In the results of Koh et al. (1992), the patella shifted laterally with knee flexion (9 mm from 0° to 60° of flexion) for a seated knee extension, while Nha et al. (2008) found a medial translation of the patella during a weight-bearing lunge exercise at low flexion angles (up to 30° of knee flexion), followed by lateral translation up to 90° of flexion. This medial to lateral movement of the patella was similar to the trend found in our results (Figure 2B). The large variability (Table 3) and scaled RMSD values of 0.7 and 0.9 for lunge and chair rise, respectively (Table 2), indicated the dependence of PML on subject and activity differences.

While several studies have presented the tibiofemoral or patellofemoral kinematics separately, few studies have focused on investigating the kinematic relationship between joint motions (Hefzy et al., 1992; Li et al., 2004; Li et al., 2007; Seisler and Sheehan 2007; Thomeer et al., 2021). In agreement with *in vitro* studies, both tibial internal rotation and patellar medial tilt increased with knee flexion (Hefzy et al., 1992). Some *in vitro* studies have shown the impact of increased tibial rotation on patellar tracking. For example, Li et al. (2004) demonstrated that increased external tibial rotation and increased posterior tibial translation raised patellofemoral contact pressures on the lateral facet of the patella. *In vivo*, Li et al. (2007) described a strong correlation between external rotation of the femur relative to the tibia (or tibial internal rotation) and patellar lateral tilt and medial translation (relative to the tibia). As both increase with knee flexion (Figures 2A,B), this correlation was also present in most of our subjects (average *R* ^2^ = 0.6, *R* ^2^ = 0.6, *R* ^2^ = 0.7 for knee extension, lunge and chair rise, respectively); however, those who utilized greater external/internal tibial rotation during their movements did not show greater patellar tilt or translation. In other words, inducing high amounts of tibial rotation may influence patellar tracking *in vitro* (Li et al., 2004), but this did not necessarily describe our subjects who naturally utilize greater tibial rotation in movement. For instance, Subjects 3 and 10 displayed very different tibial rotation and low-point kinematics (Figure 3), yet they had similar patellar kinematics. This was also true for subjects with greater anterior translation during weight-bearing who did not demonstrate greater changes in patellar kinematics. Scaled RMSD of the tibiofemoral kinematics did not correlate with the scaled RMSD of the patellofemoral kinematics, suggesting that high variability in one DOF did not indicate high variability in another.

Our results for PTA were consistent with the magnitude and trend of previous studies that measured PTA during knee extension and deep knee flexion (Yamaguchi and Zajac 1989; Miller et al., 1998; Price et al., 2004; Ali et al., 2016). In agreement with prior reports, the patellar tendon angle was positive (angled forward relative to the long axis of the tibia) with the knee extended and decreased with knee flexion (Figure 4A). Differences in PTA were small between non-weight-bearing and weight-bearing (e.g., scaled RMSD was 0.2 and 0.2 for lunge and chair rise, respectively). Even so, there were large differences in PTA between subjects, spanning from 7° to 21° at full extension (Figure 4A), which is important due to the notable influence of PTA on patellofemoral (Elias et al., 2004), tibiofemoral (Shelburne et al., 2004), and ligament loads (Carter et al., 2017).

Our results for PTMA were supported by values reported in previous studies. In Krevolin et al. (2004), the peak moment arm ranged from 40 to 60 mm and occurred at about 45° of knee flexion. Our results for PTMA also ranged from 40 to 60 mm over the same range of knee flexion but peaked between 0° and 30°. These results were more in line with the study of Ali et al. (2016) where similar trends in PTMA were observed for a simulated deep knee bend. The effect of weight-bearing on PTMA was not small for seated knee extension and lunge and like PTA, there were large differences between individuals. For instance, PTMA ranged between 45° and 60 mm at full extension (Figure 4B) across subjects. Substantial variations in PTMA can have implications for repairs that involve preservation of the quadriceps mechanism, such as total knee arthroplasty (Fitzpatrick et al., 2013), and the calculation of muscle forces using musculoskeletal models that frequently assume the same moment arm across subjects (Delp et al., 2007; Navacchia et al., 2017).

Comparison of our results to a prior work in younger healthy subjects revealed some qualitative similarities and differences. For example, TIE was greater in our study than that reported by Qi et al. (2013) for a deep knee bend, while our results were similar to those reported by Leszko et al. (2011). As described before, patellar kinematics and PTA were similar to prior work (Li et al., 2007; Thomeer et al., 2021). However, comparisons between the older adults in our cohort and younger subjects in prior work should be considered with caution due to the differences in how subjects perform high-knee flexion activities in different laboratories. Quantitative assessment of the effect of age within our cohort showed some association between age and TF and PF kinematics. However, these effects were small relative to subject variability and likely reflect the narrow distribution of age in our subject group.

There were several notable limitations of this study. The results were based on the measurement of one trial for each activity and subject. Repeated measurements may have given better results by enabling assessment of intra-subject variability; however, this was deemed unjustifiable for the additional X-ray exposure. In addition, compared to traditional motion capture, the number of subjects might be considered relatively small; however, the subject numbers were comparable to other studies of knee kinematics using similar imaging technology (Li et al., 2007; Myers et al., 2012; Thomeer et al., 2021). The number of subjects did not allow statistical examination of differences between male and female subjects. Even so, tibiofemoral kinematics between the male and female subjects was observed to be similar, although there was a trend toward greater medial tilt and lateral translation of the patella in the female subjects during weight-bearing. Our study was focused on measurements in older adults; we chose to measure older adults because the motion of younger subjects may not be representative of the age range associated with knee pain, osteoarthritis, and knee arthroplasty. Finally, using the femoral shaft proximal to the knee rather than the hip center to define the sagittal orientation of the vertical axis of the femoral coordinate system might have created a sagittal angular offset in the definition of zero flexion angle in subjects with substantial anterior bowing of the distal femur. We chose not to record the hip joint center during CT to reduce X-ray exposure.

## Conclusion

This study investigated the natural kinematics of the tibia and patella relative to the femur in healthy, older adults using a high-speed stereo radiography system. Measurements were made during a non-weight-bearing activity and two weight-bearing activities for a cohort of older adults. While weight-bearing elicited changes in knee kinematics, intersubject differences exceeded the differences observed due to weight-bearing in most DOFs. Similarly, patellar tendon angle and moment arm were consistent for the three activities but varied substantially among subjects. These results provide comparative kinematics for the evaluation of knee pathology and treatment in older adults and emphasize the need for considering subject-specific kinematics.

## Data Availability

The datasets presented in this study can be found in online repositories. The names of the repository/repositories and accession number(s) can be found at: digitalcommons.du.edu/orthopaedic_biomechanics.
